# Fibroblast growth factor 2 protects against renal ischaemia/reperfusion injury by attenuating mitochondrial damage and proinflammatory signalling

**DOI:** 10.1111/jcmm.13203

**Published:** 2017-05-24

**Authors:** Xiao‐Hua Tan, Xiao‐Meng Zheng, Li‐Xia Yu, Jian He, Hong‐Mei Zhu, Xiu‐Ping Ge, Xiao‐Li Ren, Fa‐Qing Ye, Saverio Bellusci, Jian Xiao, Xiao‐Kun Li, Jin‐San Zhang

**Affiliations:** ^1^ Key Laboratory of Biotechnology and Pharmaceutical Engineering School of Pharmaceutical Sciences Wenzhou Medical University Wenzhou Zhejiang China; ^2^ Center for Translational Medicine Department of Biotechnology Dalian Institute of Chemical Physics Chinese Academy of Sciences Dalian Liaoning China; ^3^ Department of Pediatric Surgery The Second Affiliated Hospital of Xi'an Jiaotong University Xi'an China; ^4^ Laboratory Animal Centre Wenzhou Medical University Wenzhou Zhejiang China; ^5^ Institute of Life Sciences Wenzhou University Wenzhou China; ^6^ Excellence Cluster Cardio‐Pulmonary System Justus‐Liebig University Giessen Germany

**Keywords:** fibroblast growth factor 2, High‐mobility group box 1, ischaemia‐reperfusion, acute kidney injury, mitochondrial dysfunction, inflammatory cytokine

## Abstract

Ischaemia‐reperfusion injury (I/RI) is a common cause of acute kidney injury (AKI). The molecular basis underlying I/RI‐induced renal pathogenesis and measures to prevent or reverse this pathologic process remains to be resolved. Basic fibroblast growth factor (FGF2) is reported to have protective roles of myocardial infarction as well as in several other I/R related disorders. Herein we present evidence that FGF2 exhibits robust protective effect against renal histological and functional damages in a rat I/RI model. FGF2 treatment greatly alleviated I/R‐induced acute renal dysfunction and largely blunted I/R‐induced elevation in serum creatinine and blood urea nitrogen, and also the number of TUNEL‐positive tubular cells in the kidney. Mechanistically, FGF2 substantially ameliorated renal I/RI by mitigating several mitochondria damaging parameters including pro‐apoptotic alteration of Bcl2/Bax expression, caspase‐3 activation, loss of mitochondrial membrane potential and K_ATP_ channel integrity. Of note, the protective effect of FGF2 was significantly compromised by the K_ATP_ channel blocker 5‐HD. Interestingly, I/RI alone resulted in mild activation of FGFR, whereas FGF2 treatment led to more robust receptor activation. More significantly, post‐I/RI administration of FGF2 also exhibited robust protection against I/RI by reducing cell apoptosis, inhibiting the release of damage‐associated molecular pattern molecule HMBG1 and activation of its downstream inflammatory cytokines such as IL‐1α, IL‐6 and TNF α. Taken together, our data suggest that FGF2 offers effective protection against I/RI and improves animal survival by attenuating mitochondrial damage and HMGB1‐mediated inflammatory response. Therefore, FGF2 has the potential to be used for the prevention and treatment of I/RI‐induced AKI.

## Introduction

AKI, previously known as ‘acute renal failure’, is a common clinical syndrome characterized by sudden loss of the ability of the kidneys to excrete wastes, concentrate urine, conserve electrolytes, and maintain fluid balance 1. The principal cause of AKI is hypoxia induced by I/RI, which can be caused by numerous clinical conditions, such as surgical interventions, organ transplantation, circulatory shock, toxic insults like haemorrhagic shock or sepsis and diseases such as myocardial infarction 2, 3, 4. Despite advances in preventive strategies and support measures, AKI continues to be associated with high morbidity and mortality, particularly in those admitted to the Intensive Care Unit, where in‐hospital mortality rates may exceed 50% 5. Extensive research has been carried out about the mechanism and intervention method of renal I/RI in the past decades, but the complex pathophysiology of AKI is yet to be fully understood and there remains a lack of definitively effective treatment for AKI. Current treatment is focused on maintaining renal perfusion and avoiding volume overload 6.

The destructive role of reactive oxygen species (ROS) in I/RI is well recognized. ROS are generated from several different sources, including NADPH oxidase, xanthine oxidase‐hypoxanthine, inflammatory cells, and mitochondria of parenchymal cells, as the result of ischaemia‐provoked derangement of the electron transport chain 7, 8. Overproduction of ROS by mitochondria plays a critical role in the pathogenesis of I/RI *via* loss of mitochondrial membrane potential (MMP), destabilizing the electron transport chain 9, 10, triggering mitochondrial DNA (mtDNA) injury 11, and direct damage to cellular components that can result in necrosis and apoptosis 12, 13, 14, 15, 16. Mitochondria are the main source of cellular ROS and contain a number of enzymes that convert molecular oxygen to superoxide or its derivative hydrogen peroxide (H_2_O_2_) 17. Mitochondrial processes have been demonstrated to be major events during apoptosis, and the Bcl‐2 family is involved in the alteration of MMP as well as the release of mitochondrial apoptotic factors. In addition, agents that open the mitochondrial ATP‐dependent potassium (K_ATP_) channel have been found to be effective in preventing renal injury *via* inhibition of mitochondrial DNA damage 11, 18, 19, 20, 21, 22. Therefore, therapeutic strategies aiming at inhibiting ROS production and protection of mitochondrial from oxidative damage may ameliorate renal I/R injury.

Inflammatory response is another key and integral process of I/R‐induced pathogenesis. The initial non‐immune injury to the renal parenchyma, such as ROS‐induced renal tubular cell apoptosis/necrosis, inevitably triggers an innate immune response leading to activation of inflammatory cells and cytokine secretion. The inflammation then imposes further damage to the renal parenchyma cells 23, 24. Accumulating evidence suggests that High‐Mobility Group Box‐1 (HMGB1) serves as a crucial link between the initial I/RI‐induced cell damage and the activation of inflammatory signalling cascade. Although previously known as a DNA‐binding transcription factor, HMGB1 has recently been recognized as a potent proinflammatory cytokine released from I/R injured cells. Circulating HMBG1 is capable of activating multiple cell surface receptors, including toll‐like receptors (TLRs), which act as a central mediator of inflammation through activation of NF‐κB and expression of proinflammatory cytokines 25. Targeting HMGB1/TLRs has been proposed and investigated as a therapeutic approach to protect renal tissue in various animal renal and other I/RI models with some promising results 26, 27, 28, 29.

Basic FGF2 is a member of a large family of growth factors consisting of 22 evolutionarily and structurally related proteins that signal through FGF receptors (FGFRs). FGF2 was first identified as a 146‐amino acid protein isolated from the pituitary 30. Extensive research has documented FGF2 as a pleiotropic cytokine, which exerts its effects *via* all four high affinity receptors (FGFR‐1 to −4) through a paracrine/autocrine mechanism. Exogenous FGF2 stimulates migration and proliferation of endothelial cells *in vivo* 31, has anti‐apoptotic activity and promotes mitogenesis of smooth muscle cells and fibroblasts, which induces the development of large collateral vessels with adventitia 32, 33. In certain disorders of the central nervous system (CNS), including ischaemic injury, FGF2 has also been examined for its therapeutic effects on the maintenance of Na^+^/K^+^‐ATPase activity against oxidative injury 34. The protective role of FGF2 against I/RI is best documented for myocardial infarction. FGF2 has been reported to reduce the size of the ischaemic region and ameliorates the associated symptoms in ischaemic myocardium 35, 36, 37. Using cardiac‐specific FGF‐2 overexpression transgenic mouse model, House *et al*. elegantly demonstrate the myocardial protection effect of FGF2 against I/RI, which involves its activation of both MAPK and PKC pathways 38, 39, 40. The role of endogenous FGF2 in renal I/RI and repair has also been recognized. Villanueva *et al*. first reported that FGF2 is expressed early during kidney development, re‐expressed in the regeneration phase after I/RI, and that FGF2 participates in the recovery process of I/RI by inducing an altered expression of morphogens through FGFR2 41, 42, 43. Despite the prominent role of FGF2 in I/RI pathogenesis and repair processes, the molecular mechanism underlying FGF2 signalling‐mediated protection against I/RI remains incompletely understood, and whether exogenous FGF2 can deliver therapeutic benefit towards renal I/RI is unknown. In studies of the brain and heart, some evidence indicates that FGF2 can modulate oxidative stress caused by the formation of ROS 37, 44. Our previous studies have demonstrated that excessive mitochondrial ROS production plays an important role in renal I/RI 6, 11. Herein, we show that either pre‐ or post‐I/R administration of FGF2 protects against renal I/RI by attenuating multiple mitochondrial damage parameters, as well as HMGB1/TLR2‐mediated proinflammatory response.

## Materials and methods

### Reagents and antibodies

Bovine serum albumin (BSA), recombinant human FGF2, sodium pentobarbital, 5‐hydroxydecanoate (5‐HD) and mitochondria isolation kits were purchased from Sigma‐Aldrich (St Louis, MO, USA). 5,*5*′,*6*,*6′‐Tetrachloro‐1*,*1′,3*,*3*′‐ tetraethylbenzimidazolylcarbocyanine iodide (JC‐1) and 4′, 6‐diamidino‐2‐phenylindole (DAPI) were purchased from Invitrogen (Carlsbad, CA, USA). Rat HMGB1 ELISA Kit was purchased from CUSABIO (Hubei, China). Antibodies against 8‐hydroxy‐2‐deoxyguanosine (8‐OHdG), phospho‐FGFR, TNFα and caspase‐9 were purchased from Abcam (Cambridge, MA, USA). Anti‐3‐Nitrotyrosine (3‐NIT) antibody was purchased from Invitrogen. Anti‐Kir6.2 antibody was purchased from Santa Cruz Biotechnology (Santa Cruz, CA, USA). Antibodies against the voltage‐dependent anion channel (VDAC), cleaved caspase‐3, HMGB1 and GAPDH were purchased from Cell Signaling Technology (Beverly, MA, USA). The secondary antibodies were purchased from Abcam or Santa Cruz Biotechnology.

### Animals and renal I/RI model

Male Sprague‐Dawley rats (SD rats, 8–10 weeks old) were purchased from Shanghai SLAC Laboratory Animal Co., Ltd. and were housed in our SPF facility under standard conditions of temperature and 12 hrs light/dark cycle with *ad libitum* feeding. The Institutional Animal Ethical Committee and Use Committee of Wenzhou Medical University approved the animal research protocol. Rats were positioned on a homoeothermic surgical platform after being anaesthetized with an intra‐peritoneal (i.p) injection of 25 mg/kg sodium pentobarbital and underwent right nephrectomy. Renal ischaemic condition was achieved by renal artery clamping for 45 min. (50 min. for survival studies) and then renal blood flow was re‐established. Kidneys were harvested 2 days after artery ischaemia and stored at −80°C until further analysis. Serum creatinine (Cr) and Blood Urea Nitrogen (BUN) were measured 2 days following renal ischaemia by the hospital laboratory. Rats were divided into four groups: (*i*) Sham‐operated animals with an unrestricted renal artery; (*ii*) I/R animals: kidneys were subjected to 45 min. of ischaemia followed by reperfusion; (*iii*) I/R+FGF2 Group, rats were treated with single dose of 0.5 mg/kg FGF2 (i.p) 1 hr before ischaemia and then subjected to 45 min. of ischaemia followed by reperfusion; (*iv*) I/R+FGF2 + 5‐HD Group, animals were treated with 0.5 mg/kg FGF2 (i.p) 1 hr before ischaemia and then treated with 5 mg/kg 5‐HD (i.m) 15 min. before ischaemia. The lyophilized recombinant FGF2 was freshly dissolved in sterile saline before use. 5‐HD was dissolved in saline. For post‐I/R treatment, rats were treated with one dose of 0.5 mg/kg FGF2 (i.p) at 1, 3, or 12 hrs after reperfusion as indicated. Twenty rats from each group were used for survival assessments.

### Histology and pathological scoring of the renal tubules

Kidney tissues were fixed in 10% formaldehyde, embedded in paraffin, and sectioned for haematoxylin and eosin (H&E) staining. Renal pathological changes were observed using light microscopy. To measure the pathological score of the renal tubules, 12 visual fields from each section were selected randomly under the microscope using previously reported methods 45, 46. Based on the assessment of tubular expansion, cast formation, brush border loss, and epithelial cell necrosis, the following 5‐point scoring system was used to assess renal pathology: 0 point (normal kidney morphology without damage); 1 point (necrosis of the renal tubules ≤10%); 2 points (necrosis of the renal tubules 11–25%); 3 points (necrosis of the renal tubules 26–45%); 4 points (necrosis of the renal tubules 46–75%); 5 points (necrosis of the renal tubules ≥76%). The pathologists were blinded to rat allocation group.

### Immunohistochemistry staining (IHC)

Kidneys were excised and harvested 2 days following 45 min. of ischaemic condition. Paraffin‐embedded sections (5 μm) were stained with H&E. Slides were incubated with anti‐FGFR antibody (1:200) at 4°C overnight and stained with diaminobenzidine tetrahydrochloride (DAB) and counterstained with haematoxylin. Oxidative damage was detected using anti‐8‐OHdG antibody (1:100) and antibody against nitrotyrosine (1:200). For caspase‐3 and caspase‐9 staining, slides were incubated with anti‐cleaved caspase‐3 antibody (1:200) and anti‐cleaved caspase‐9 antibody (1:200), respectively. Apoptosis was detected using a one‐step TUNEL Apoptosis Assay KIT (Roche, Mannheim, Germany) according to the manufacturer's instructions. Slides were also counterstained with DAPI at 37°C for 5 min. to identify nuclei. The images were captured with a Nikon ECLIPSE Ti microscope (Nikon, Melville, NY, USA). The percentage of positive cells with dual TUNEL and DAPI staining at five randomly selected 400× fields served as the index of apoptosis. For Kir6.2 and VDAC double staining, sections were incubated with goat anti‐Kir6.2 antibody (1:200) and mouse anti‐VDAC antibody (1:200) at 4°C overnight and then with fluorescein isothiocyanate‐labelled donkey anti‐goat IgG (1:200) and phycoerythrin‐labelled donkey antimouse IgG (1:200) for 60 min. Cell nuclei were counterstained blue with DAPI at 37°C for 5 min. Sections were analysed by fluorescence microscopy.

### Western blot analysis

For protein analysis of *in vivo* samples, renal tissues were dissected, snap frozen and stored at −80°C. Protein extracts from renal tissues were prepared by centrifuging the complex mixed 0.1 g kidney with protein extraction reagents and subjected to Western blot. Protein concentrations were measured with a BCA Protein Assay Kit. Equal amounts of protein were loaded into lanes and separated on SDS‐PAGE, followed by transfer to a polyvinylidene fluoride membrane. After blocking in 5% skim milk in Tris‐buffered saline/0.1% Tween‐20 (TBST), the membrane was incubated with following antibodies against Kir6.2, Bcl‐2, Bax, 3‐Nit, cytochrome C, caspase‐3, VDAC and GAPDH, respectively. After washing with TBST three times, membranes were incubated with secondary antibodies for 1 hr at room temperature. The signals were visualized with the ChemiDoc XRS+Imaging System (Bio‐Rad Laboratories, Hercules, CA, USA). The band densities were quantified with Multi Gauge Software of Science Lab 2006 (FUJIFILM Corporation, Tokyo, Japan).

### Determination of MMP

We measured MMP with freshly isolated mitochondria and paraffin‐embedded sections (5 μm). Kidney tissue was homogenized in 50 mM Tris‐HCl buffer (pH7.4) and centrifuged at 2000×*g* in 4°C for 5 min to precipitate the nuclear fraction. The supernatant was then centrifuged at 11,000×*g* in 4°C for 10 min. to yield the mitochondrial fraction. The mitochondrial pellet was suspended in 40 μl of storage buffer. Mitochondria protein and paraffin sections were incubated with 1 μg/ml JC‐1 for 10 min. at 37°C according to the manufacturer's instructions. The electrical potential across the inner mitochondrial membrane (Δψ) was detected using the laser confocal at an excitation wavelength of 485 nm and an emission wavelength of 590 nm.

### ELISA

Serum HMGB1 was measured using ELISA kit (CUSABIO) according to the manufacturer's instructions. The result was expressed as pg/ml. The lower detection limit was 62.5 pg/ml.

### Real‐time quantitative RT‐PCR

Total RNA was isolated from kidney using RNeasy column (QIAGEN, Germantown, MD, USA), reverse transcribed using PrimeScript™ RT reagent Kit (TaKaRa, Berkeley, CA, USA) according to the manufacturer's instructions. Real‐time PCR was performed using the SYBR Green gene expression assays (TaKaRa) for detection of mRNA expression levels. The PCR primers used for mRNA expression analysis for GAPDH, KIM1, TLR2, TLR4, IL‐Iα, IL‐6 and TNFα are shown in Table 1. The target values were normalized to GAPDH.

**Table 1:** Primers used to amplify rat cDNAs

Gene	GenBank	Primer sequences
GAPDH	NM_012675	5′‐ GACATGCCGCCTGGAGAAAC‐3′ 5′‐AGCCCAGGATGCCCTTTAGT‐3′
IL‐1β	NM_031512	5′‐TGCAGGCTTCGAGATGAAC‐3′ 5′‐GGGATTTTGTCGTTGCTTGTC‐3′
IL‐6	NM_012589	5′‐AAGCCAGAGTCATTCAGAGC‐3′ 5′‐GTCCTTAGCCACTCCTTCTG‐3′
KIM‐1	NM_173149	5′‐CTCTGTTGATAGTGATAGTGGTCTG‐3′ 5′‐TGTGGGTCTTGTAGTTGTGG‐3′
TLR2	NM_198769	5′‐ATGAACACTAAGACATACCTGGAG‐3′ 5′‐CAAGACAGAAACAGGGTGGAG‐3′
TLR4	NM_019178	5′‐CATGACATCCCTTATTCAACCAAG‐3′ 5′‐GCCATGCCTTGTCTTCAATTG‐3′
TNFα	NM_012675	5′‐CTTCTCATTCCTGCTCGTGG‐3′ 5′‐TGATCTGAGTGTGAGGGTCTG‐3′

IL‐1β: interleukin‐1β; IL‐6: interleukin‐6; TLR2: Toll‐like receptor‐2; TLR‐4: Toll‐like receptor‐4; KIM1: Kidney Injury Molecule‐1; TNFα: tumour necrosis factor‐α; GAPDH: Glyceraldehyde 3‐phosphate dehydrogenase.

### Statistical analysis

SPSS 19.0 statistical software (Cary, NC, USA) was used for data analysis. The Kaplan–Meier method was used to compare the survival rates. Data are expressed as the mean ± S.E.M. of *n* independent experiments. When more than two groups were compared, statistical evaluation of the data was performed using one‐way analysis of variance (anova). Tukey multiple comparison was used as a *post hoc* analysis. A value of *P* < 0.05 was considered statistically significant.

## Results

### FGF2 pre‐treatment attenuates I/RI‐induced renal dysfunction and pathologic damage

**Figure 1:**
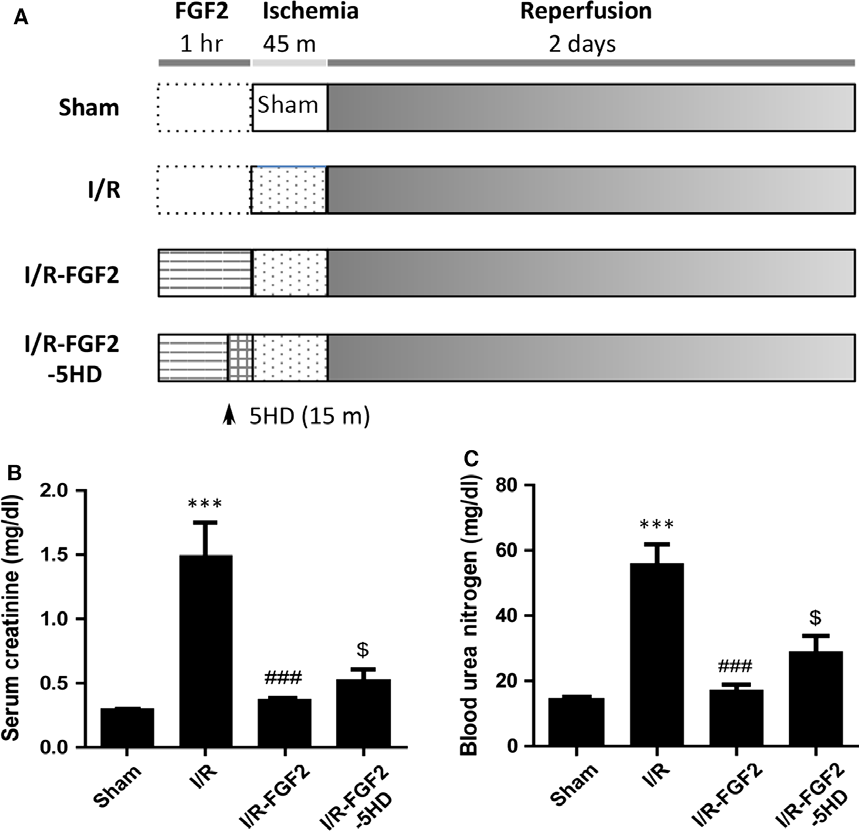
FGF2 sustains renal function after I/R injury. (**A**) Protocol for renal ischaemia/reperfusion injury model in rat. (**B**) Determination of serum Cr levels in indicated animal groups at 48 hrs after reperfusion (mean ± S.E.; *n* = 8). ***P < 0.001 *versus* Sham group, ###*P* < 0.001 *versus* I/R group; $*P* < 0.05 *versus* FGF2 + I/R group. (**C**) Determination of blood urea nitrogen (BUN) levels in indicated animal groups at 48 hrs after reperfusion (mean ± S.E.; *n* = 8). ***P < 0.001 *versus* sham group; ###*P* < 0.001 *versus* I/R group; $*P* < 0.05 *versus* FGF2 + I/R group.

**Figure 2:**
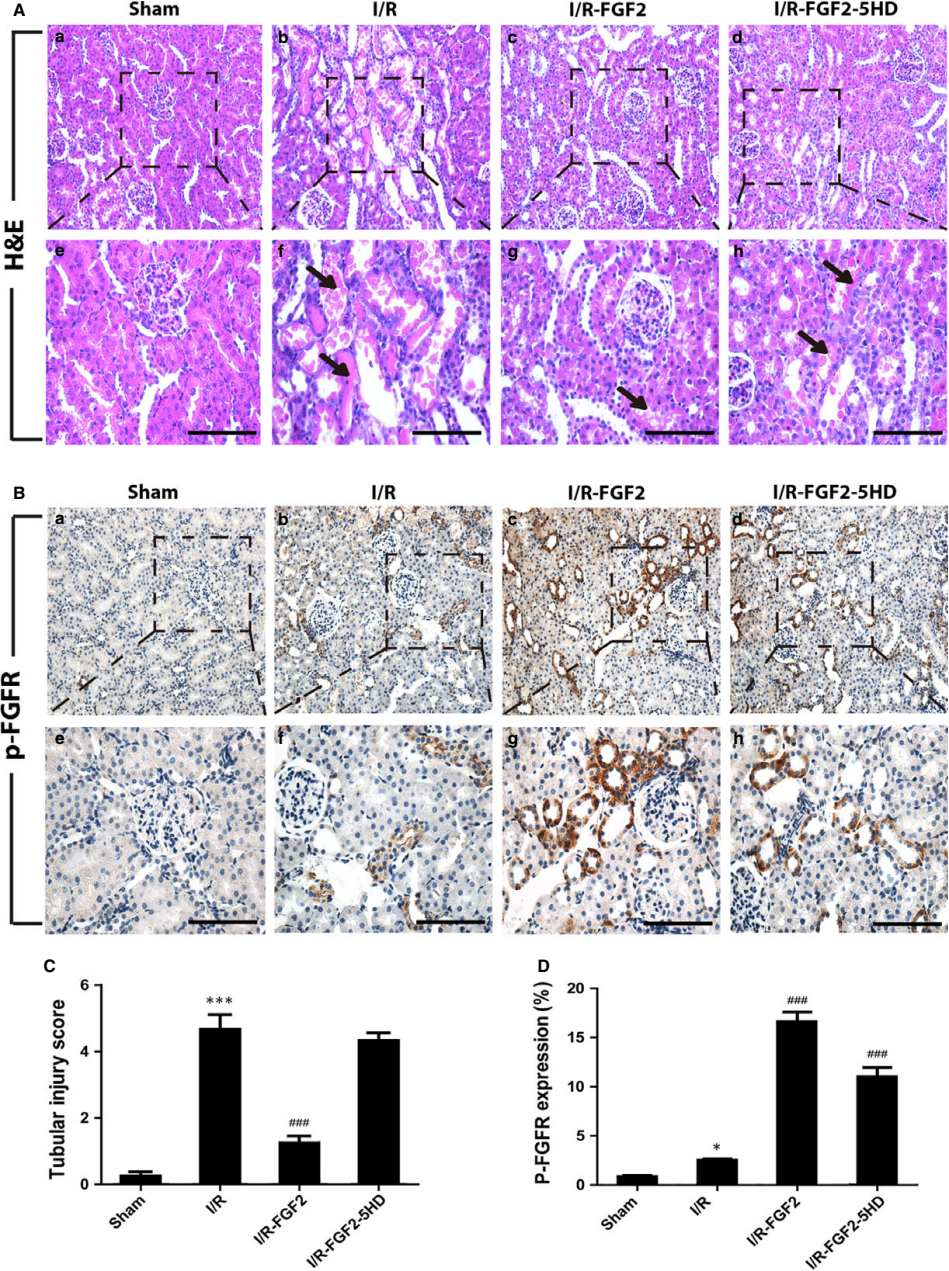
FGF2 protects renal histological integrity and robustly activates FGFR. (**A**) Histological evaluations of renal tissue with H&E after 2 days of reperfusion (original magnification ×20 and ×40, respectively). Arrows show intraluminal necrotic cellular debris, interstitial congestion and oedema, and formation of proteinaceous casts. Scale bars represent 50 μm. (**B**) Immunohistochemical staining for phospho‐FGFR (p‐FGFR, original magnification ×20 and ×40, respectively). Scale bars represent 50 μm. (**C**) Renal tubular injury scores were calculated based on H&E staining using the criteria and procedure described in the material and methods. Results are representative of eight animals in each group. ***P < 0.001 *versus* sham group), ###*P* < 0.001 *versus* I/R group. Results are representative of eight animals in each group. (**D**) Quantification and statistical analysis of p‐FGFR positive cells in the kidney. Data are representative of five animals in each group. *P < 0.05 *versus* sham group.

To assess the histology of AKI and protective effect of FGF2 on renal function, we employed a rat model of I/R‐induced AKI (Fig. 1A). Two days following I/R, the serum levels of Cr and BUN were significantly higher in I/R rats compared with sham‐ operated rats (Fig. 1B and C). Strikingly, serum levels of both Cr and BUN in I/R+FGF2 rats were much lower compared to I/R rats (*P* < 0.001) and revealed no significant difference to the sham‐operated rats, whereas 5‐HD treatment partially reversed the action of FGF2. We next evaluated the histopathological changes in the kidney tissue 2 days after reperfusion. Representative H&E stained kidney sections from each group are shown in Figure 2A. No significant damage was observed in the kidney sections from rats of the sham group (Fig. 2A‐a, e). In contrast, the I/R group displayed typical features of AKI characterized by cellular swelling, intraluminal necrotic cellular debris, vacuolar degeneration, luminal narrowing, interstitial congestion and oedema, and formation of proteinaceous casts (Fig. 2A‐b, f). Quantification analysis of renal damages indicated that a near 15‐fold increase in tubular injury score in I/R group as compared to sham control (*P* < 0.001), whereas FGF2 pre‐treatment significantly lowered the score compared to, an effect largely blunted by 5‐HD co‐treatment (Fig. 2C). Consistent with the reduced levels of serum Cr and BUN, FGF2 administration markedly reduced kidney tissue damage compared to the I/R group (Fig. 2A‐c, g), whereas 5‐HD significantly antagonized the protection activity of FGF2 (Fig. 2A‐d, h).

### FGF2 treatment enhances I/RI‐induced activation of endogenous FGFR

To investigate the relationship between FGF2/FGFR pathway and renal I/RI, we determined the activation status of FGFR by IHC staining of kidney tissue sections with anti‐ p‐FGFR antibody. Very few p‐FGFR and weak positive renal tubular epithelial cells were present in kidneys of sham rats (Fig. 2B‐a, e), while this number is perceptibly increased 2 days after reperfusion (Fig. 2B‐b, f). However, both the number of p‐FGFR positive cells and the staining intensity were markedly increased in renal tubules of I/R kidney with FGF2 alone or combined with 5‐HD (Fig. 2B‐c, d, g, h) compared to sham group and I/R alone. Quantification analysis shown in Figure 2D indicated that the number of p‐FGFR positive cells was already significantly increased by I/RI (*P* < 0.05), whereas FGF2 treatment led to more robust increase (*P* < 0.001).

### FGF2 pre‐treatment protects the renal tubular cells from I/R‐induced apoptosis

**Figure 3:**
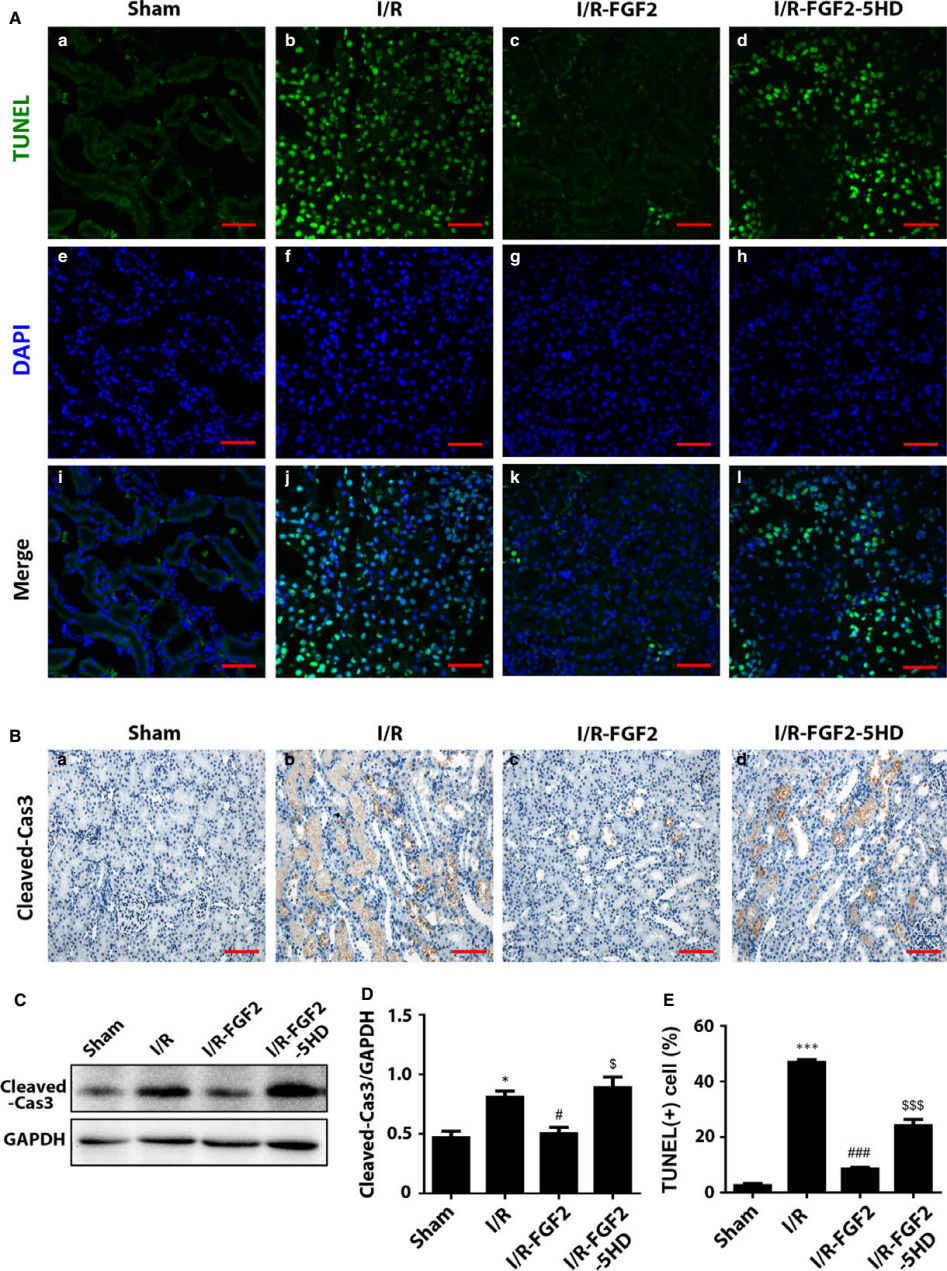
FGF2 protects renal tubular cells from I/R‐induced apoptosis. (**A**) Representative sections of nuclear DNA fragmentation after 2 days of reperfusion. Staining was achieved by TdT‐mediated dUTP nick‐end labelling (TUNEL) immunofluorescence. Original magnification ×40, scale bars represent 50 μm. Results are representative of five animals in each group. (**B**) IHC staining for Cleaved caspase‐3 (Original magnification ×40, scale bars represent 50 μm). Data are representative of five animals in each group. (**C**) Western blot analyses of Cleaved caspase‐3 expression. GAPDH was used as a loading control. Representative data of three individual samples per group. (**D**) The optical density analysis of Cleaved caspase‐3 in the kidney. *P < 0.05 *versus* sham group, #*P* < 0.05 *versus* I/R group; $*P* < 0.05 *versus* I/R+FGF2 group. (**E**) The percentage of TUNEL‐positive cells was counted from five random 1 mm^2^ areas. Data are presented as the mean ± S.D. ***P < 0.001 *versus* sham group; ###*P* < 0.001 *versus* I/R group; $$$*P* < 0.001 *versus* I/R+FGF2 group.

TUNEL staining of kidney tissue sections revealed a small number of TUNEL‐positive tubular epithelial cells were present in the sham rats (Fig. 3A‐a). Consistent with I/RI‐induced apoptotic phenotype, the number of TUNEL‐positive cells was dramatically increased after 2 days of reperfusion (Fig. 3A‐b). Importantly, FGF2 pre‐treatment largely prevented the I/RI‐induced apoptotic cell death (Fig. 3A‐c), whereas co‐treatment with 5‐HD significantly reduced the FGF2 protection effect (Fig. 3A‐d). To determine the possible pathway of I/R injury, we performed IHC staining of activated caspase‐3 and observed that its expression was significantly increased in kidneys of I/R group and I/R+FGF2+5‐HD group, but was dramatically lower in I/R+FGF2 group (Fig. 3B). This result was confirmed by Western blot, which indicated that the expression of cleaved caspase‐3 was significantly decreased in I/R+FGF2 kidney tissues compared with I/R or I/R+FGF2+5‐HD groups (Fig. 3C–E). The results indicated that FGF2 administration exerted potent renal protective effects against I/R‐induced apoptosis by reducing the caspase activation.

### FGF2 pre‐treatment alters the expression of key mitochondrial apoptosis‐regulatory proteins

**Figure 4:**
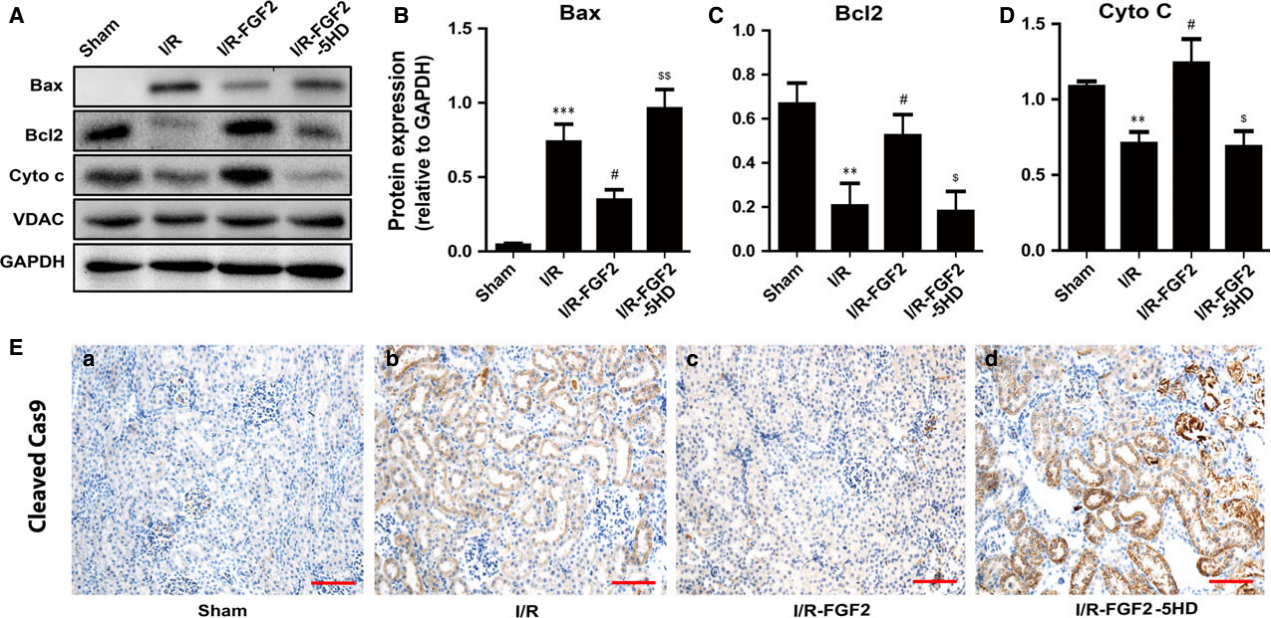
FGF2 ameliorates pro‐apoptotic mitochondrial protein expression. (**A**) Immunoblot analysis of mitochondrial damage‐related proteins in the kidneys 2 days after reperfusion. (**B‐D**). Optical density analysis to quantify protein expression levels for Cytochrome C (Cyto‐C), Bax and Bcl2 in kidneys of sham rats, I/R rats, I/R+FGF2 rats and I/R+FGF2 + 5‐HD rats (mean ± S.E.; *n* = 5). **P < 0.01 and ***P < 0.001 *versus* sham group, #*P* < 0.01 *versus* I/R group, $*P* < 0.05 and $$*P* < 0.01 *versus* FGF2 + I/R group. (**E**) IHC staining of Cleaved caspase‐9 (original magnification ×20, scale bars represent 100 μm). Data are representative of five animals in each group.

To determine whether the renal protective effect of FGF2 is related to its ability to maintain mitochondrial integrity following I/RI, we measured the expression of several key mitochondrial proteins involved in apoptosis by immunoblot. Observation revealed that the level of Bax expression was significantly up‐regulated in renal tissues of I/R rats compared with sham control (Fig. 4A and B). However, increased Bax expression was partially inhibited by FGF2 pre‐treatment. In contrast, the protein levels of Bcl‐2 and cytochrome c in mitochondria (Cyto‐c) were significantly down‐regulated in the kidneys of I/R rats compared with sham rats. Importantly, FGF2 treated animals did not show a significant loss of either Bcl‐2 or Cyto‐C compared with sham group (Fig. 4A, C, D). IHC analysis indicated that FGF2 inhibited the up‐regulation of cleaved caspase‐9 that was induced by I/RI (Fig. 4E).

### FGF2 pre‐treatment greatly alleviates I/RI‐induced mitochondrial oxidative damage

**Figure 5:**
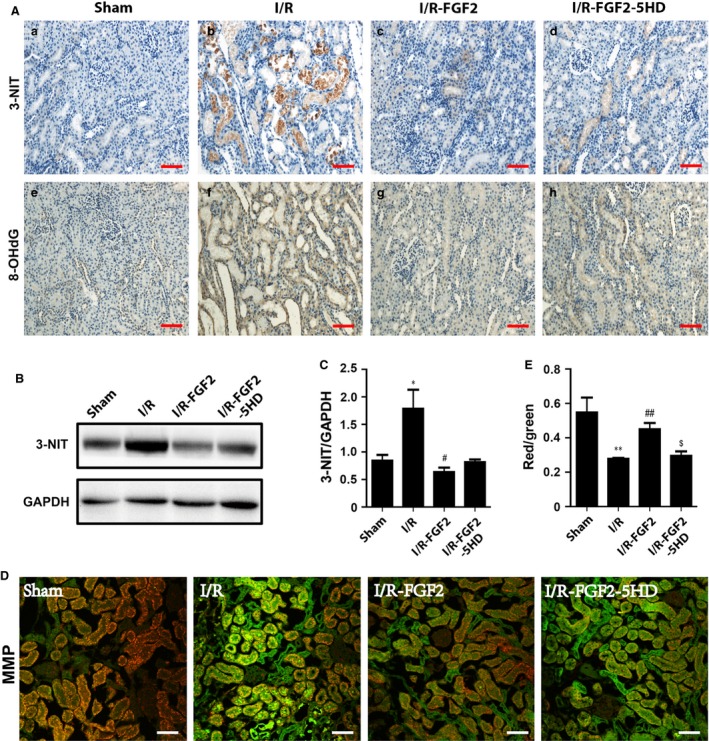
FGF2 alleviates I/R‐induced mitochondrial oxidative damage. (**A**) Immunohistochemistry staining for 3‐nitrotyrosine and 8‐OHdG 2 days after reperfusion. Results show positive staining of 3‐nitrotyrosine and 8‐OHdG primarily localized in tubular epithelial cells. FGF2 treatment reduced 3‐nitrotyrosine and 8‐OHdG to levels similar to sham rats. Original magnification ×20, scale bars represent 100 μm. Renal tissue sections from 1 of 4 animals in each group are shown. (**B**) Western blot analysis of 3‐nitrotyrosine expression in the kidney. GAPDH was used as a loading control. (**C**) Optical density analysis to quantify protein expression for 3‐nitrotyrosine in the kidney (mean ± S.E.; *n* = 4). *P < 0.05 *versus* sham group, #*P* < 0.05 *versus* I/R group. (**D**) Detection of mitochondrial membrane potential (MMP) in kidney using the JC‐1 MMP detection Kit and confocal microscope imaging analysis. MMP declined in I/R kidney after 2 days of reperfusion as indicated by JC‐1 fluorescence shift from red towards green, a phenomenon reversed by FGF2 treatment. Original magnification ×20, scale bars represent 100 μm. Data are representative of five animals in each group. (**E**) MMP in freshly isolated kidney mitochondria was also measured by the JC‐1 MMP detection Kit. **P < 0.01 *versus* sham group, ##*P* < 0.01 versus I/R group, $*P* < 0.05 *versus* I/R+FGF2 group.

Nitrotyrosine immunohistochemistry staining was performed to reveal peroxynitrite formation caused by mitochondrial oxidative damage. I/RI increased nitrotyrosine production after reperfusion, as demonstrated by strong tubular epithelial cell staining of kidney tissue sections (Fig. 5A‐b). The production of nitrotyrosine was significantly lower in I/R+FGF2 kidneys when compared with I/R kidneys (Fig. 5A‐c). Immunoblot analysis of nitrotyrosine is consistent with IHC staining as I/R‐induced nitrotyrosine accumulation is largely abolished by FGF2 (Fig. 5B and C). It is well accepted that mtDNA is more susceptible than nuclear DNA to increase oxidative stress due to the lack of histone protection 47. MtDNA damage caused by oxidative stress can be assessed staining with 8‐OHdG staining. Indeed, we detected increased production of 8‐OHdG in the cytoplasm of tubular cells in ischaemic kidneys by IHC (Fig. 5A‐f), while FGF2 treatment inhibited the production of 8‐OHdG (Fig. 5A‐g). Of note, staining of 8‐OHdG was primarily localized in the cytoplasm, indicating that this oxidative adduct was mainly present in the mitochondria.

### FGF2 pre‐treatment mitigates the loss of MMP following I/RI

Mitochondria isolation kit (Sigma‐Aldrich) was used to prepare the mitochondria containing intact inner and outer membranes 11, 48. MMP was detected in paraffin‐embedded sections 2 days after reperfusion by use of the fluorescent probe JC‐1 (Invitrogen). We observed that relative fluorescence intensity of red to green was lower in I/R kidneys compared with the sham control, whereas relative fluorescence intensity of red to green was contrastively higher in I/R+FGF2 kidneys compared to I/R kidneys (5D). We also measured the MMP using JC‐1 in freshly isolated mitochondria 2 days after reperfusion. Consistent with results of measurements in tissue sections, the intensity of red fluorescence was reduced almost half in I/R kidneys compared with sham control. The lack of significant difference in MMP between I/R+FGF2 and sham control (Fig. 5E) suggests that FGF2 could contribute in maintaining a near homoeostatic MMP; which may be essential for the functional integrity of mitochondria and cell survival 49.

### FGF2 pre‐treatment contributes to sustain mitochondrial K_ATP_ channel upon I/RI

**Figure 6:**
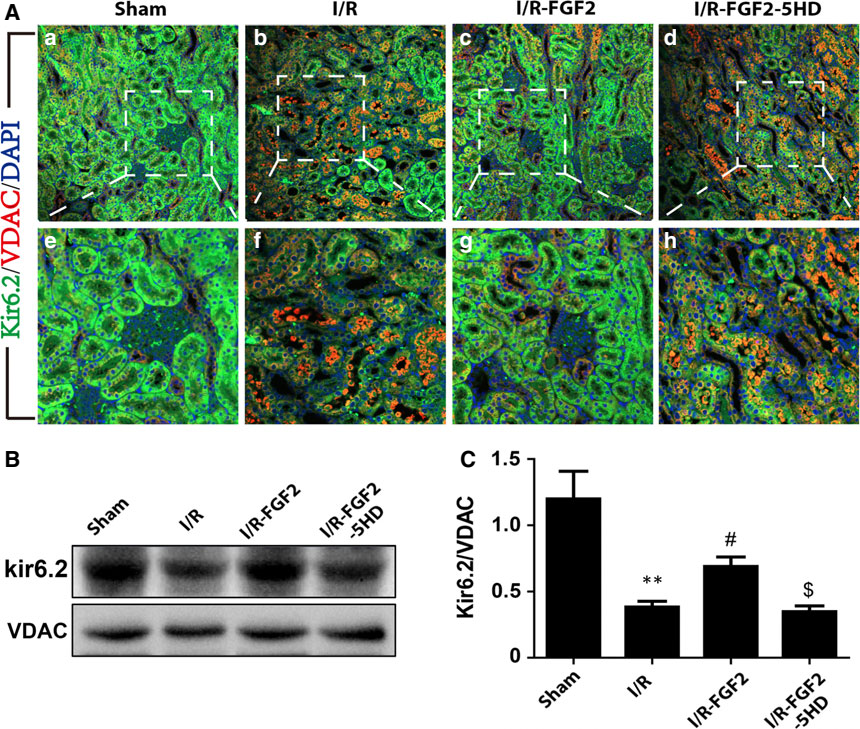
FGF2 contributes to maintain mitochondrial K_ATP_ channel expression and functional integrity. (**A**) Expression of mitochondrial ATP‐dependent potassium (K_ATP_) channel subunit Kir6.2 was determined by immunofluorescence staining 2 days after reperfusion. Kir6.2 (in green) was widely distributed in renal tubular epithelial cells and was more abundant than VDAC (in red) in sham‐operated kidney, but Kir6.2 expression declined dramatically in I/R animals, which was largely reversed by FGF2 treatment. The effect of FGF2 in reversing the decrease of Kir6.2 expression is counteracted by 5‐HD co‐treatment. Results are representative of four animals from each group. (**B**) Western blot analysis of Kir6.2 protein expression. VDAC was used as an internal control. FGF2 treatment sustained Kir6.2 expression, but this effect was reversed by 5‐HD (mean ± S.E.; *n* = 4). **P < 0.01 *versus* sham group, #*P* < 0.05 *versus* I/R group, $*P* < 0.05 *versus* FGF2 + I/R group. (**C**) The optical density analysis of Kir6.2 in the kidney tissue sections.

Previous studies have shown that Kir6.2, a subunit of the mitochondrial K_ATP_ channel, is localized to the mitochondria of renal tubular epithelial cells, smooth muscle cells and cardiomyocytes 50, 51. To determine whether FGF2 treatment influenced mitochondrial K_ATP_ channels, subunit Kir6.2 was examined by immunofluorescence staining, using VDAC as an internal control. Immunofluorescence staining showed that Kir6.2 expression (green fluorescence) was decreased in ischaemic kidneys after 2 days of reperfusion. However, FGF2 treatment sustained Kir6.2 expression and this effect was reversed in the 5‐HD treatment (Fig. 6A). Western blot analysis confirmed that the decrease of Kir6.2 expression relative to VDAC (Kir6.2/VDAC) was largely prevented upon FGF2 treatment of I/R kidneys, whereas 5‐HD treatment abolished this protection effect (Fig. 6B and C).

### Delayed FGF2 treatment exerts equal protection against renal I/RI

**Figure 7:**
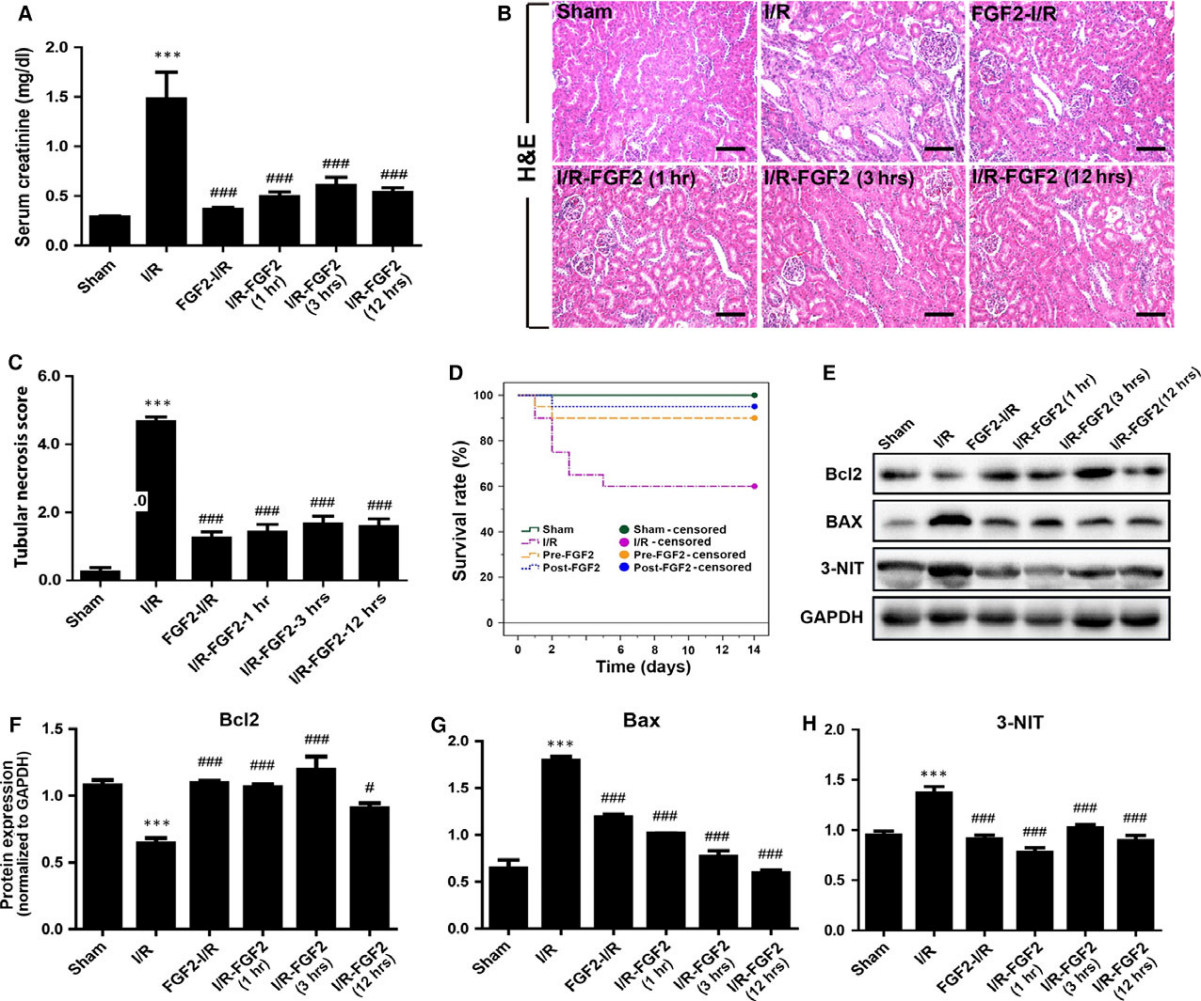
Delayed FGF2 treatment exhibits potent protection against I/RI. Animals were divided into six groups, including sham‐operated control, I/RI group, and I/RI rat with FGF2 pre‐treatment or post‐I/R treatment at 1, 3 and 12 hrs, respectively, after reperfusion as indicated. Except for the animal survival analysis, which was carried out for 2 weeks, all other experiments were performed at 48 hrs after reperfusion. (**A**) The serum creatinine (Cr) levels of animals receiving indicated treatment, including sham‐operated (sham), ischaemia‐reperfusion (I/R), I/R pre‐treated with FGF2 (FGF2‐I/R), or I/R with delayed FGF2 treatment at 1, 3, or 12 hrs, respectively, after reperfusion as indicated. ***P < 0.001 *versus* sham group, ###*P* < 0.001 *versus* I/R group. (**B**) Representative H&E stained renal tissue sections. Original magnification ×20, scale bars represent 100 μm. (**C**) Histological evaluation and pathological scoring based on H&E staining. Renal tubular necrosis scores were calculated using the criteria and procedure described in the material and methods. Results are representative of eight animals in each group. ***P < 0.001 *versus* sham group), ###*P* < 0.001 *versus* I/R group. (**D**) Effect of FGF2 treatment on the survival of I/R rats. Twenty rats were assigned to each group as indicated and animal survival curves (Kaplan–Meier analysis) were constructed at 14 days after reperfusion. Upon 50 min. of ischaemic exposure, the survival rate of I/RI rats was 60% compared to 100% in sham‐operated control. Notably, FGF2 pre‐treatment (Pre‐FGF2) or delayed treatment (Post‐FGF2, 12 hrs after reperfusion) resulted in significantly increased animal survival rates (90% and 95%, respectively. *P* < 0.05 *versus* I/R alone group). There was no significant difference between pre‐ and post‐FGF2 group. (**E**) Renal protein extracts were subjected to Western blot analysis with indicated antibodies to determine the expression of Bcl2, Bax, 3‐NIT with GAPDH as loading control. (**F–H**) Optical density analysis to quantify the expression levels of Bax, Bcl‐2 and 3‐NIT, respectively, with indicated treatments. The data were shown as mean ± S.E. (*n* = 5). ***P < 0.001 *versus* sham group, ###*P* < 0.001 and #*P* < 0.05 *versus* I/R group.

To explore the potential of FGF2 for therapy, we further investigated the effect of delayed FGF2 administration on renal I/RI and repair. Animals were treated with FGF2 at 1, 3 and 12 hrs, respectively, post renal ischaemic exposure besides the pre‐I/R treatment as above. We assessed and compared the degree of renal dysfunction in each group by measuring their serum creatinine levels. Serum Cr measurement indicated that post‐I/R FGF2 treatment at all three time‐points displayed similar and marked alleviation of renal functional impairment to the pre‐I/R treatment compare to sham‐treated group (Fig. 7A). H&E staining of renal tissue sections and quantification analysis of tubular injury score indicated that post‐I/R FGF2 was equally effective in preserving the renal histology as pre‐I/R administration (Fig. 7B and C). The potent effect of exogenous FGF2 against I/R‐induced functional impairment and histology damage prompt us to determine whether FGF2 would bring any survival benefit to the injured animals. Kaplan–Meier analysis revealed that no mortality was observed in the rats from the sham‐operated group, whereas sham‐treated I/R animals exhibited a survival rate of 60% with most of fatalities occurring within 3 days of reperfusion. Importantly, both the pre‐I/R and 12 hrs post‐I/R FGF2 treatment significantly improved the animal survival of I/R rats (Fig. 7D). Consistent with the protection against ROS damage and apoptosis, post‐I/R FGF2 also induced Bcl2 expression, while markedly decreased the levels of both Bax and 3‐NIT expression. Overall these results indicate that post‐I/R administration of FGF2 equally protects kidney from I/R‐induced mitochondrial damage and improves animal survival.

### FGF2 inhibits I/RI‐induced HMGB1 release and inflammatory cytokine gene expression

**Figure 8:**
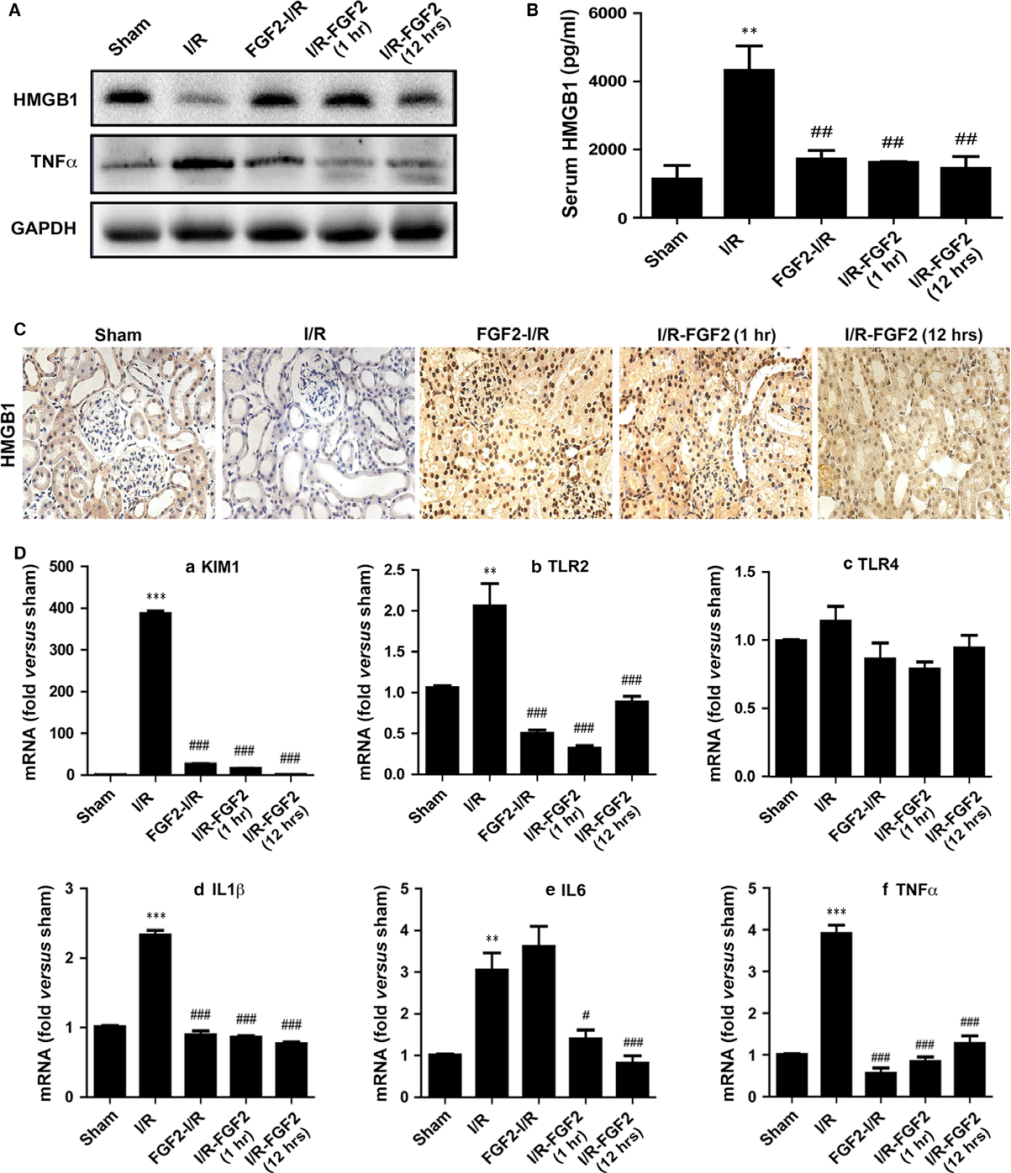
FGF2 inhibits I/RI‐induced HMGB1 serum release and inflammatory response. Animals were divided into 5 groups (*n* = 4), including sham‐operated control, I/RI group, and I/RI with FGF2 pre‐treatment or delayed treatment at 1 and 12 hrs, respectively, after reperfusion as indicated. The samples were collected at 48 hrs following reperfusion for Western blot, ELISA, Immunohistochemistry staining (IHC) and qRT‐PCR analysis as detailed below. (**A**) Western blot analysis to determine the expression of HMGB1 and TNFα in renal tissues with GAPDH as loading control. (**B**) ELISA assay was used to determine the levels of HMGB1 in the serum of animals receiving indicated treatments. **P < 0.01 *versus* sham group, ##*P* < 0.01 *versus* I/R group. (**C**) IHC of kidney tissue sections for expression of HMGB1. Original magnification ×20. One representative area of renal tissue staining from 1 of 4 animals in each group is shown. (D) Real‐time PCR quantification of mRNA levels for KIM1, TLR2, TLR4, IL‐1α, IL‐6 and TNFα in the kidney, respectively. The result is normalized to GAPDH. The data are presented as mean ± S.E. (*n* = 4). ***P < 0.001, **P < 0.001 *versus* sham group; ###*P* < 0.001, #*P* < 0.05 *versus* I/R group.

HMGB1 is a major damage‐associated molecular pattern (DAMP) molecule released by damaged cells upon I/RI, which contributes to proinflammatory signalling to inflict broader tissue damage. To determine whether FGF2 could affect the nuclear–cytoplasmic trafficking and extracellular release of HMGB1 in I/R kidneys, we first assessed the expression of HMGB1 by immunoblot analysis. HMGB1 is readily detected in protein extract of sham‐operated kidney (Fig. 8A), but was markedly decreased upon I/RI. Significantly, either pre‐ or post‐I/R exposure to exogenous FGF2 blunted the I/R‐induced decrease of HMGB1. TNFα, a key downstream inflammatory cytokine, was significantly induced in I/R kidney, and conversely largely prevented by FGF2 treatment (Fig. 8A). Contrary to the decrease in kidney tissue, serum HMGB1 level, as measured by ELISA, was increased to nearly fourfold in the I/R group. Notably, either 1 or 12 hrs post‐I/R given FGF2 achieved similar levels of inhibition towards HMGB1 release to the serum (Fig. 8B). IHC staining further confirmed the HMGB1 release following ischaemia (Fig. 8C). Compared to the sham control (Fig. 8C‐a), HMGB1 staining intensity was greatly reduced in I/R kidney (Fig. 8C‐b), with minimal nuclear HMGB1 left in the tubular cells. Strikingly, either pre‐ or post‐I/R FGF2 treatment effectively preserved the intracellular level of HMGB1, particularly its nuclear localization (Fig. 8C‐c, d, e). These results together indicate that either pre‐ or post‐IR FGF2 prevented I/RI‐induced HMGB1 nucleus to cytoplasm translocation and extracellular release.

Extracellular HMGB1 activates proinflammatory response mainly *via* activation of cell surface receptors including TLRs. TLR2 and TLR4, in particular, are essential in mediating renal I/R‐induced damage and inflammatory cytokine gene expression, such as IL1β, IL‐6 and TNFα. We next measured the mRNA expression of these inflammatory factors *via* real‐time PCR. As expected, the expression of Kidney Injury Molecule‐1 (KIM‐1), a biomarker for renal proximal tubule injury, was dramatically increased upon I/RI with up to a nearly 400‐fold induction over the sham‐operated control (Fig. 8D‐a). Consistent with its protective effect, FGF2 treatment dramatically reduced I/RI‐induced KIM1 expression (Fig. 8D‐a). In the same cDNA samples, TLR2 expression was increased twofold in the I/R group, which was completely prevented by either Pre‐ or post‐ischaemic FGF2 treatment (Fig. 8D‐b). We did not observe significant change with TLR4 expression in any of the I/R groups regardless of FGF2 treatment (Fig. 8D‐c). The expression for IL‐1β, IL‐6 and TNFα all showed significant increases in I/RI kidney relative to sham control (Fig. 8D‐d, e, f). Importantly, FGF2 treatment effectively blunted the I/R‐induced expression of these five pro‐inflammatory genes. The only exception is IL‐6, for which the I/R‐induced expression was not inhibited by pre‐ischaemic FGF2 (Fig. 8D‐e). The data together strongly indicate that FGF2 exerts potent inhibitory activity towards I/R‐induced HMGB1 release and the expression of key proinflammatory genes, such as IL‐1β, IL‐6, TLR2 and TNFα.

## Discussion

The molecular mechanism of I/RI remains incompletely understood and there are no definitive treatment measures for I/RI‐induced AKI 52. Current work demonstrates that I/R‐induced renal tubular cell apoptosis is associated with pro‐apoptotic alteration of key mitochondria protein (Bcl2 and Bax) expression, decreased MMP and caspase activation. The accumulation of oxidative mtDNA damage in this I/R‐induced AKI model is also evident and likely contributes to the mitochondria dysfunction and apoptosis. Strikingly, FGF2 treatment dramatically alleviated the various damaging parameters of I/RI, including oxidative mtDNA damage and decrease in MMP. Furthermore, the reduced expression of the mitochondrial K_ATP_ channel subunit Kir6.2 following I/R was largely rescued in FGF2 treated animals. Nonetheless, blocking of K_ATP_ channels with the classic mK_ATP_ inhibitor 5‐HD reduced the protective effect of FGF2. Therefore, FGF2 appears to act upon several key aspects of mitochondrial function to mediate its protection effect.

A number of previous studies including those from our own group have reported the protective effect of FGF2 against I/RI in myocardial, retinal, Intestinal and neural tissues in various experimental models 34, 35, 36, 37, 41. However, the protective mechanism of FGF2 on renal I/RI model remains unclear. In this study, we found that both renal functional and morphological integrity were largely preserved in I/R kidney group receiving pre‐ or post‐ischaemic FGF2 treatment. Interestingly FGF2 has previous been identified as an I/R‐induced nephrogenic protein with the inhibition of FGFR2 by antisense oligonucleotides enhancing ischaemic AKI damage, thus suggesting that endogenous FGF2/FGFR2 signalling is involved in the renal I/RI repair process. To determine how effectively FGF2 activated the FGFRs in this model, we further analysed the expression of activated FGFR using a phospho‐FGFR antibody. Consistent with the reported increase of FGF2 following I/RI, we observed mild and localized activation of FGFR in proximal renal tubular cells, whereas FGF2 administration robustly enhanced the level of phospho‐FGFR compared with control I/R animals. Therefore our experimental data combined with the published work strongly suggest that the protective effect of FGF2 towards renal I/RI is mediated through its activation of FGFRs.

It is well‐established that apoptosis plays an important role in renal I/R pathogenesis 53. The Bcl‐2 family of anti‐apoptotic proteins has a major role in maintaining the integrity of the external mitochondrial membrane and preventing the release of cytochrome C from the mitochondria. Conversely, several pro‐apoptotic proteins such as Bax promote mitochondrial injury and cytochrome C release into cytoplasm from cell mitochondria. Released cytochrome C combined with apoptotic protease activating factor‐1 (Apaf‐1) and caspase‐9 produce the Cytc‐Apaf‐1‐Caspase‐9 complex. This leads to the activation of pro‐caspase‐9 and subsequent downstream activation of caspase‐3, which initiates the apoptotic cascade leading to cells death *via* either apoptosis or necrosis 54, 55. To address if FGF2 has an impact on AKI‐induced apoptotic process and how it may affect the apoptotic regulatory proteins, we first demonstrated that exogenous FGF2 dramatically reduced the number of TUNEL‐positive tubular cells and decreased the activation of caspase‐3, as well as caspase‐9 in renal tubular cells compared with I/R alone (Fig. 3B). Western blot analysis revealed that the levels of mitochondrial cytochrome C and cytoplasmic Bcl‐2 were increased. On the contrary, cytoplasmic Bax expression induced by I/RI was significantly decreased by FGF2 treatment (Fig. 4A). Together our results suggest that FGF2 prevents I/RI‐induced apoptosis in a mitochondrial dependent mechanism *via* regulation of Bcl2 family of proteins.

Generation of ROS by mitochondria also contributes to damage of cellular components and initiation of cell death. MtDNA is more susceptible than nuclear DNA to increased oxidative stress because of the lack of histone protection and limited capacity of DNA repair capability 47, 56. 8‐OHdG is a biomarker of oxidative DNA damage, which stains nuclear DNA as well as mtDNA. Nitrotyrosine, a marker of nitrosative stress, was increased in renal tubular epithelial cells after I/R. ROS reacts with nitric oxide generating peroxynitrite, which may bind to protein residues such as tyrosine and yield highly cytotoxic nitrotyrosine 57, 58. In our previous study, we demonstrate that ROS, the major initiator of lethal effects of I/R injury, were rapidly produced in the mitochondria of renal tubular cells following reperfusion. Agents that open the mitochondrial K_ATP_ channel reduced the generation of ROS by the mitochondria in as early as 1 hr post reperfusion 11. However, whether FGF2 can protect damage to mtDNA had not been previously investigated. In the current study, protection of mtDNA by FGF2 was reflected by lowering the amounts of 3‐NIT generation and less mtDNA oxidative damage when compared with those in I/R rats (Fig. 5A and B). We have previously proposed that mtDNA damage may be the cause of renal injury and could occur even before cell death 11. To determine whether mtDNA damage indeed influenced mitochondrial function, we further measured MMP with an immunofluorescence‐based assay. MMP was significantly decreased 2 days after reperfusion; however, FGF2 treatment largely sustained MMP level in the treated animals (Fig. 5D and E).

K_ATP_ channels have a critical role in maintaining cellular energetic homoeostasis under physiological conditions, highlighted by the fact they sustain MMP during oxidative stress 59. Opening of the K_ATP_ channel has been proposed to be associated with an uptake of potassium in the mitochondrial matrix, which could constitute a parallel potassium influx and attenuate Ca2^2+^ overload. The reduction in mitochondrial Ca^2+^ uptake would prevent mitochondrial swelling and inhibit opening of the mitochondrial permeability transition pore during reperfusion 60. Additionally, mitochondrial K_ATP_ channel activity effectively inhibits the development and release of ROS 61, which are the reactive molecules and possible initiators of all deleterious effects seeing following reperfusion. A number of studies have concluded that activation of mitochondrial K_ATP_ channels confer protection against I/RI, which has been shown not only by pharmacological means using mitochondrial K_ATP_ channel activators and inhibitors, but also by direct evidence of Kir6.2 gene overexpression 62, 63, 64.

We speculated that the protective mechanisms of FGF2 were related to its effect on mitochondrial K_ATP_ channels. To test this hypothesis, 5‐HD, an ischaemia‐selective mitochondrial K_ATP_ antagonist 65, was administered before ischaemia. 5‐HD was chosen, due to its acceptance as a more specific blocker of mitochondrial K_ATP_ channel than glibenclamide 66. In our present study, Kir6.2 expression was significantly decreased in renal tubular epithelial cells 2 days after reperfusion, while FGF2 treatment resulted in significant up‐regulation of Kir6.2 expression, which was completely antagonized by 5‐HD (Fig. 6). We speculate that opening of mitochondrial K_ATP_ channels may be a protective mechanism of FGF2; therefore, blocking of mitochondrial K_ATP_ channels blunted its protection effect.

The innate immune response is another key pathogenic aspect of I/RI. The initial hypoxic damage to parenchymal cells, particularly the highly susceptible proximal tubular cells, inevitably triggers sterile inflammatory response contributing to the infliction of broad tissue damage. HMGB1 has been identified as a major DAMP protein capable of imposing potent proinflammatory response once released by damaged cells. Thus far, we are unaware of any published research on I/RI linking FGF2 to regulation of inflammatory response. By combining analysis of immunoblot, ELISA and IHC of the same samples, we obtained convincing evidence that I/R‐associated cytoplasmic translocation and extracellular release of HMGB1 can be completely inhibited by either pre‐I/R or delayed FGF2 treatment. As circulating HMGB1 is known to activate proinflammatory signalling pathways through its interaction with pattern recognition receptors such as TLR2 and TLR4; both of which are crucial pro‐inflammatory factors in exacerbating I/RI as demonstrated using their respective knockout mouse models 45, 67. We therefore further measured the mRNA expression of TLR2 and TLR4 in the kidneys, and confirmed that I/RI significantly induced TLR2 expression, which is completely obliterated upon FGF2 treatment. Contrary to the earlier report on I/RI‐induced TLR4 expression, we found TLR4 to be only marginally induced in the same samples measured at 48 hrs post‐I/R exposure. This result was confirmed by testing two independent sets of primers with the same result (Fig. 8D‐c, and data not shown). Consistent with the potent inhibitory effect of FGF2 treatment on I/R‐induced HMGB1 release and TLR2 expression, we affirmed complete obliteration, by FGF2 treatment, of I/R‐induced IL‐1α, IL‐6 and TNFα expression, which are three key inflammatory cytokines downstream of HMGB1/TLRs signalling pathway. Therefore, current work provides the first experimental evidence that FGF2‐mediated protection against I/RI involves its ability to mitigate subsequent inflammatory responses. Whether this potent anti‐inflammatory effect of FGF2 is due to its protection of renal parenchymal cells from initial hypoxic damage, alleviating the extracellular release of HMGB1 and subsequent proinflammatory response, or additional mechanism involving direct interaction FGF2/FGFR signalling with the immune cells will be an interesting topic for future study.

From the clinical translational point of view, FGF2 has been successfully used as a repair/regeneration factor in a variety of conditions such as burns, chronic wounds, oral ulcers, vascular ulcers, diabetic ulcers, pressure ulcers and surgical incisions. To date all of these indications have been limited to topical application 68. However, a number of recent studies including those from our group have reported the protective effect of FGF2 against I/R injuries in various disease models; particularly in cardiac infarction 36, 37, 38, 39, 40. The current work investigated the effect of both pre‐I/R and delayed FGF2 administration and demonstrated its potent protection against I/R‐induced mitochondrial damage and anti‐inflammatory response. Given that delayed FGF2 treatment at 12 hrs post‐IR still showed significant protective activity and improved animal survival, our findings further highlight the potential of FGF2 in the prevention and treatment of I/R‐induced AKI. As the prototype member of the FGF family, FGF2 is known to engage all four FGFRs and activate signalling pathways including Ras‐MAPK and PI3K/AKT pathways, which contribute to cell proliferation and survival. Indeed, in several ischaemic disease models such as the cardiac infarction, activation of MAPK, PI3K/AKT, or PKC signalling pathway by FGF2 is essential to mediate its protection 38, 39, 40. The fact that FGF2 administered at 12 hrs post‐I/R still delivered significant protection strongly suggests a role of FGF2 in the repair process of AKI in addition to its aid in the protection from initial damage. It has been reported that exogenous FGF2 promotes cardiac stem cell‐mediated myocardial regeneration and enhance the viability of cord blood‐derived mesenchymal stem cells transplanted to ischaemic limbs 69, 70. Further study is required to delineate the role as well as the molecular mechanism of FGF2 on renal tissue repair and regeneration through stem cell differentiation and/or mobilization.

In summary, current study provides the first experimental evidence that exogenously administered FGF2 exhibits robust protection against renal I/RI and significantly improves animal survival. The remarkable protective effect pertains to its ability to attenuate several I/RI‐induced mitochondrial‐damaging parameters including pro‐apoptotic alteration of Bcl2/Bax expression and caspase‐3 activation. FGF2 also contributes to maintain the expression of mitochondrial K_ATP_ channels under hypoxic condition, which may help to alleviate oxidative stress and I/R‐induced mtDNA damage. Beside the above protection on renal parenchyma, FGF2 treatment is also capable of obliterating I/R‐triggered HMGB1 release and activation of TLRs‐mediated signalling and inflammatory cytokine gene expression. These new mechanistic insights into FGF2‐mediated protection against renal I/RI may also shed light to understand I/RI‐related disorders in other tissues. Given that either pre‐ischaemic or post‐I/R FGF2 treatment delivers similar potency of protection, our work suggests that FGF2 has the potential to be used for the prevention as well as treatment of I/RI‐related AKI.

## Conflicts of interest

The authors confirm that there are no conflicts of interest.
